# Short-term memory of temporal information revisited

**DOI:** 10.1007/s00426-020-01343-y

**Published:** 2020-04-28

**Authors:** Daniel Bratzke, Rolf Ulrich

**Affiliations:** 1 University of Bremen, Hochschulring 18, 28359 Bremen, Germany; 2 University of Tübingen, Tübingen, Germany

## Abstract

Previous studies provided diverging evidence regarding modality specificity of temporal information in short-term memory. Some authors reported modality-specific interference effects on visual and auditory duration discrimination, whereas others observed crossmodal interference effects. One reason for these diverging results could be different trade-offs between the temporal discrimination task and the interference task in these studies. Therefore, this study re-examined these effects with interference tasks (speeded color/pitch change discrimination) that were especially suited to assess potential trade-offs between the primary and the secondary tasks. The results showed that the auditory interference task selectively impaired discrimination performance for auditory durations, whereas the visual interference task proved to be inefficient as interference task. The present results agree best with an account that suggests a modality-specific representation of temporal information in short-term memory.

## Introduction

How is temporal information encoded and maintained in short-term memory? Contemporary timing theories imply three different hypotheses regarding this question. First, internal clock models (Creelman, 1962; Gibbon, Church, & Meck, 1984; Treisman, 1963) suggest an abstract and amodal representation (Rammsayer & Ulrich, 2005; Wearden, Todd, & Jones, 2006), that is, the number of signals elicited by an internal pacemaker during a certain time interval represent the duration of this interval. This amodal hypothesis appears especially plausible because humans (and animals) can easily compare durations not only within sensory modalities but also across modalities. Second, temporal information might be primarily encoded in the auditory system (crossmodal encoding; Bratzke, Seifried, & Ulrich, 2012; Guttman, Gilroy, & Blake, 2005; Kanai, Lloyd, Bueti, & Walsh, 2011), because the auditory system is especially suited for temporal processing (Welch & Warren, 1980). Third, intrinsic timing models imply that the short-term representation of temporal information is modality-specific because temporal processing is an inherent feature of early sensory processing (Buonomano & Karmarkar, 2002; Ivry & Schlerf, 2008).

An important source of evidence regarding these hypotheses comes from studies investigating modality-specific interference effects on short-term memory of temporal information. In one of these studies, Rattat and Picard (2012) presented unimodal (visual vs. auditory) and bimodal (visual-auditory) durations. To load the visual and the auditory short-term memory sub-systems (i.e., the visuo-spatial sketchpad and the phonological loop; e.g., Baddeley & Hitch, 1974) during an 8-s retention interval, they used a visuo-spatial tracking task and articulatory suppression, respectively. In a control condition, participants simply waited for the retention interval to elapse. After the retention interval, participants’ memory for the duration was probed by a comparison stimulus that could be shorter or longer than the initial stimulus. The results showed that visual tracking selectively impaired short-term memory of visual durations, and articulatory suppression selectively impaired short-term memory of auditory durations. The authors interpreted this selective interference pattern as evidence for a modality-specific representation of duration information.

Bratzke, Quinn, Bausenhart, and Ulrich (2016) partially replicated the study by Rattat and Picard (2012), focusing on the unimodal conditions and employing a within- instead of a between-subjects design. In contrast to Rattat and Picard, Bratzke et al. observed that articulatory suppression impaired short-term memory not only for auditory but also for visual durations. Additionally, visual tracking did not interfere with retention of the duration information, neither for visual nor for auditory stimuli. This result pattern is consistent with the crossmodal and the amodal encoding view (an amodal code is maintained in the phonological loop), but not with the modality-specific view.

How can the discrepancy between these two result patterns be explained? Bratzke et al. (2016) identified several methodological differences between the two studies (usage of fixation point, performance feedback, different ranges of visual comparison durations, within- vs. between subjects design), of which, however, none provided a particularly plausible explanation for the different results. Another possibility is that the different results reflect different trade-offs between the primary timing task (duration discrimination task) and the secondary interference tasks (visuo-spatial tracking and articulatory suppression) in different conditions of the two studies. For example, in the study by Rattat and Picard (2012), participants might have preserved their performance in the visual duration discrimination task by putting less emphasis on the articulatory suppression task. Neither Rattat and Picard nor Bratzke et al. registered performance in the interference tasks (Bratzke et al.’s participants were video-monitored without recording) so that this possibility cannot be evaluated based on their previous results.

**Fig. 1:**
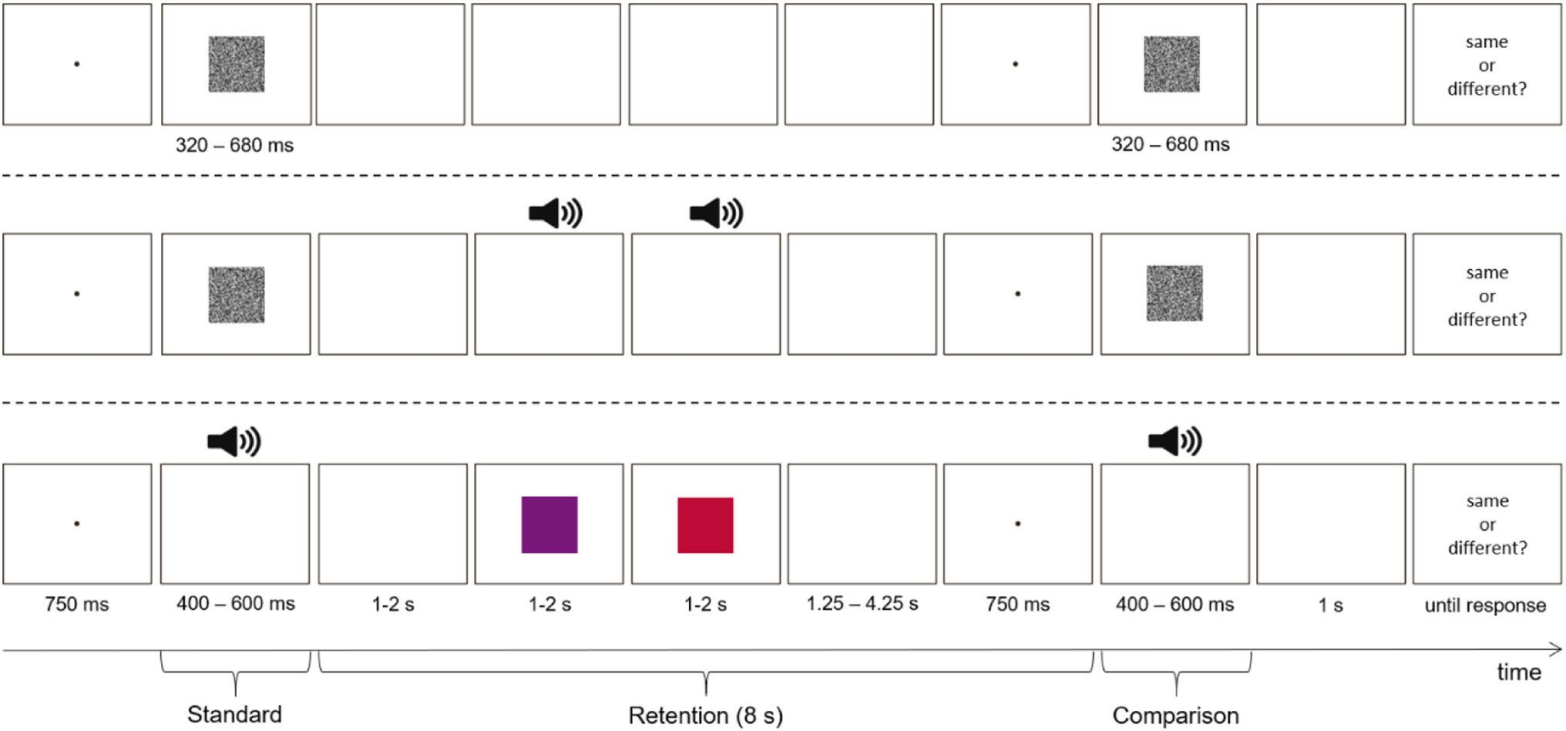
Time course of three exemplary experimental trials. Upper panel: visual duration discrimination with no interference. Middle panel: visual duration discrimination with auditory interference (pitch discrimination). Lower panel: auditory duration discrimination with visual interference (color discrimination)

In the present study, we aimed to reassess interference effects between a primary duration discrimination task and secondary non-temporal interference tasks and control for possible trade-offs between primary and secondary task performance. Since measuring and analyzing performance in the visual tracking and the articulatory suppression task is rather costly (i.e., it requires eye tracking and video monitoring), we used interference tasks that allow a more efficient way of measurement. The visual interference task (color discrimination) was adopted from Klauer and Zhao (2004). In their study, this task proved to be effective in interfering with short-term retention of unfamiliar Chinese ideographs. The auditory interference task was designed to resemble the visual interference task (pitch discrimination). The time-course of a single trial followed the two previous studies (Bratzke et al. 2016; Rattat & Picard, 2012), that is, participants had to discriminate two duration stimuli, a standard and a comparison, separated by an 8-s retention interval, which was either empty or filled with an interference task (see Fig. 1). As in the two previous studies, visual and auditory duration discrimination were tested in different sessions, and within each session, the interference tasks were tested in different blocks. As in Bratzke et al. we employed a within-subjects design, that is, all participants performed all conditions.

## Method

### Participants

The final sample consisted of 48 participants (36 women) with a mean age of 23.5 (SD = 3.6) years. Each participant took part in two separate sessions of the experiment (each lasting about 45 min). They received either course credit or payment for their participation. Eight original participants were replaced by new participants because they did not adhere to the instructions or achieved an accuracy in the interference tasks at or below chance level (≤ 53% correct).

### Apparatus and stimuli

The experiment was programmed in MATLAB^®^ using the Psychophysics Toolbox extension (Brainard, 1997; Kleiner et al., 2007). Participants sat in front of a computer screen with a viewing distance of approximately 50 cm. Auditory stimuli were presented binaurally via headphones (Sennheiser HD 380 pro) with a peak amplitude of 60 dB (A) SPL. White noise (400, 440, 480, 520, 560, and 600 ms) with rise and fall times of 5 ms served as the auditory duration stimulus in the duration discrimination task. In the pitch discrimination task, there was a reference sine tone of 800 Hz and 24 test sine tones ranging from 320 to 1280 Hz (in steps of 40 Hz with the exception of 800 Hz).

Visual stimuli were presented on a 21-inch CRT monitor (Samsung SyncMaster 1100 MB, 1024 × 768 pixels, 150 Hz). To make the visual duration stimuli as similar as possible to the auditory ones, we used a square (11.4 × 11.4° of visual angle) filled with dynamic grey visual noise (random grey pattern of 256 × 256 pixels with 150 Hz refresh rate) as the visual duration stimulus. As in Bratzke et al. (2016), we used a wider range of durations for the visual (320, 380, 440, 560, 620, and 680 ms) than for the auditory stimuli (400–600 ms) to compensate for the typically observed poorer temporal sensitivity for visual than auditory stimuli (e.g., Bratzke & Ulrich, 2019b; Grondin, 1993; Ulrich et al., 2006). A violet-colored (RGB values: 122, 24, 122) square of the same size (11.4 × 11.4° of visual angle) served as reference stimulus in the color discrimination task. There were 24 test colors, of which 12 can be categorized as more reddish or bluish than the reference color. The RGB values of the reddish stimuli ranged from (190, 11, 55) to (135, 22, 110) and those of the bluish stimuli ranged from (110, 22, 135) to (55, 11, 190), with steps of five units for the *R* and *B* values and 1 unit for the *G* value. All visual stimuli were presented at the center of the screen on a white background. Responses were collected using the ‘X’ (left index finger) and ‘M’ (right index finger) keys on a standard German keyboard.

### Procedure

Each participant took part in two experimental sessions, a visual and an auditory discrimination session (with a maximum separation of one week between the two sessions). Each trial started with the presentation of a small fixation point at the center of the screen. After 750 ms, the standard duration was presented, chosen randomly with equal probability from the six possible durations. Then, an 8-s retention interval started with a blank screen. 750 ms before the end of the retention interval the fixation point reappeared and remained at the center of the screen until the presentation of the comparison duration. After the retention interval, the comparison duration was presented. This stimulus was either equal to the standard duration (50% “same” trials) or it differed (50% “different” trials). In “different” trials, when the standard duration was shorter than 500 ms, the comparison duration was 120 (240) ms longer in auditory (visual) trials. When the standard duration was longer than 500 ms, the comparison duration was 120 (240) ms shorter in auditory (visual) trials. 1000 ms after the end of the comparison duration, a prompt message was presented asking the participant to indicate whether the durations of the two stimuli were equal or different. The message included a reminder of the response-to-key key assignment in the duration discrimination task. The next trial started 1000 ms after the participant’s response. For the duration discrimination task, participants were instructed to respond as accurately as possible without any speed restrictions.

Within each modality session (visual vs. auditory duration discrimination), participants completed three blocks of trials, each differing in the nature of the interference task presented during the retention interval. In “no interference” trials, the screen remained empty during the first 7.25 s of the retention interval. In color discrimination trials (visual interference), the reference-colored square appeared after a random interval between 1 and 2 s from the start of the retention interval. After another random interval between 1 and 2 s, the square changed its color to one of the 24 test colors. Participants had to indicate whether the color changed toward more reddish or bluish. Irrespective of the participant’s response time, the test color stimulus remained on the screen for another random interval between 1 and 2 s. Pitch discrimination trials (auditory interference) followed the same time course as visual ones. In these trials, first the reference tone was played, which then changed to one of the 24 possible test tones. Participants had to indicate whether the tone’s pitch changed toward higher or lower. For the interference tasks, participants were instructed to respond as quickly and as accurately as possible to the change of the reference stimulus.

For each of the combinations of duration modality (visual vs. auditory) and interference task (no interference, color discrimination, and pitch discrimination), participants completed three practice trials (randomly chosen from all possible trials within each factorial combination) and four experimental blocks of 12 trials each (one “same” and one “different” trial for each of the possible 6 durations). Within each experimental block, all durations were presented equally often and the order of trials was randomized. After each experimental block, participants received feedback about the percentage of correct responses in the duration discrimination task in the preceding block. Duration modality varied between sessions, and interference task varied between blocks within each session. The order of modality sessions and interference tasks was balanced across participants. The response-to-key assignment in the duration discrimination task was also balanced across participants. The response-to-key assignment in the interference tasks was the same for all participants (color discrimination: more bluish—‘X’/more reddish—‘M’; pitch discrimination: lower—‘X’/higher—‘M’).

## Results

As in Bratzke et al. (2016), trials in which RT for the duration discrimination task exceeded 5 s were regarded as lapses and discarded from all analyses (1.0% of all trials). As in previous studies (Bratzke et al., 2016; Rattat & Picard, 2012), for the duration discrimination task the dependent variables were the nonparametric indices $A^{\prime}$ and $B^{\prime\prime}$ for sensitivity and bias (Aaronson & Watts, 1987; Grier, 1971), respectively. Trials in which participants gave no response in the interference task before the end of the retention interval (0.4% of remaining trials) were excluded from these analyses. For the interference tasks, mean RTs (of correct interference trials) and error rates were calculated. Separate ANOVAs with the within-subjects factors duration modality (visual vs. auditory) and interference task (no interference, color discrimination, and pitch discrimination) were conducted for $A^{\prime}$, $B^{\prime\prime}$ in the duration discrimination task, and for mean RT and error rate in the two interference tasks. The Greenhouse–Geisser correction was used to adjust *p* values where appropriate.

### Primary task performance (duration discrimination)

**Fig. 2:**
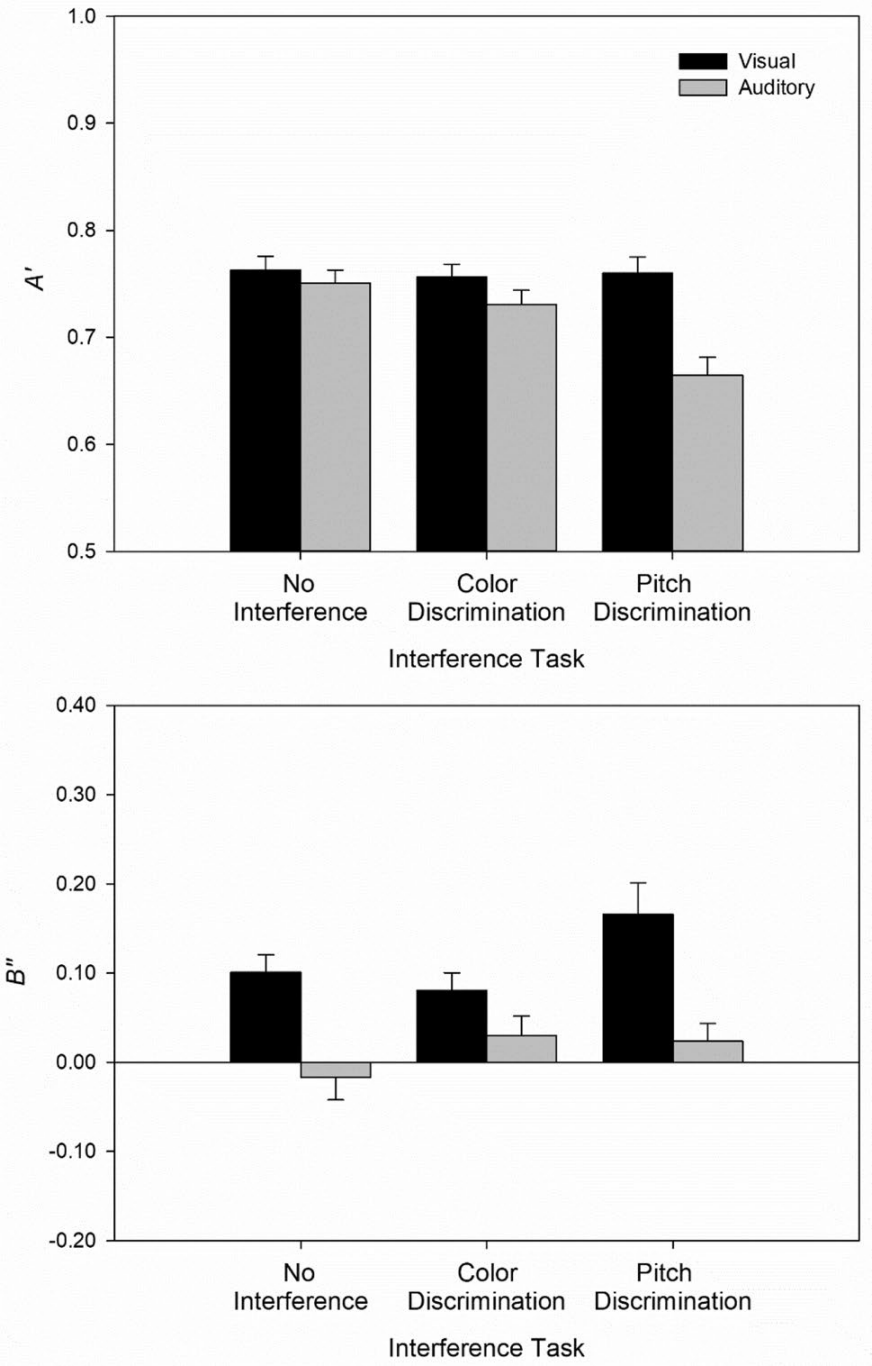
Sensitivity *A*′ (upper panel) and bias *B*″ (lower panel) as a function of interference task and duration modality. Error bars represent  ± 1 within-subject error according to Morey (2008)

Figure 2 depicts $A^{\prime}$ (upper panel) and $B^{\prime\prime}$ (lower panel) values as a function of duration modality and interference task. Note that $A^{\prime}$ can range from 0 to 1, with $A^{\prime}$ = 0.5 indicating chance level, and $A^{\prime}$ = 1 representing perfect discrimination performance. ANOVA revealed a significant main effect of interference task, *F*(2, 94) = 5.56, *p* = 0.005, ${\eta }_{p }^{2}= 0.11$. Post hoc Tukey contrasts indicated a reliable difference between pitch discrimination and no interference (0.71 vs. 0.76), *p* = 0.006, a marginally significant difference between pitch and color discrimination (0.71 vs. 0.74), *p* = 0.077, and no significant difference between color discrimination and no interference (0.74 vs. 0.76), *p* = 0.639. This pattern suggests that only pitch discrimination interfered with short-term memory of temporal information. The main effect of duration modality was also significant, *F*(1, 47) = 11.06, *p* = 0.002, ${\eta }_{p }^{2}= 0.19$. Discrimination performance was on average slightly better for visual (0.76) than for auditory (0.72) duration stimuli. A paired sample *t-*test on discrimination performance only in the control condition revealed no difference between visual and auditory stimuli (0.76 vs. 0.75), *t*(47) = 0.63, *p* = 0.531. Most important, the interaction between duration modality and interference task was also significant, *F*(2, 94) = 6.43, *p* = 0.002, ${\eta }_{p }^{2}= 0.12$. Separate ANOVAs for the two duration modalities revealed that the interference tasks impaired discrimination performance when the duration stimuli were auditory, *F*(2, 47) = 11.02, *p* < 0.001, ${\eta }_{p }^{2} = 0.12$, but not when the stimuli were visual, *F*(2, 47) = 0.07, *p* = 0.934, ${\eta }_{p }^{2}< 0.01$. Post-hoc Tukey contrasts indicated that pitch discrimination differed from color discrimination (*p* = 0.002) as well as no interference (*p* < 0.001), and that color discrimination did not differ from no interference (*p* = 0.567). Together, these results show a selective interference pattern: only the auditory interference task (pitch discrimination) impaired discrimination performance and this was only the case for auditory duration stimuli.

In contrast to the previous results by Bratzke et al. (2016) and Rattat and Picard (2012), most of the $B^{\prime\prime}$ values were slightly positive (see lower panel of Fig. 2), indicating a small bias towards “different” responses ($B^{\prime\prime}$ values can range from − 1 to + 1, with negative values indicating a bias towards “same” responses and negative values indicating a bias towards “different” responses). There was a significant main effect of duration modality, *F*(1, 47) = 20.97, *p* < 0.001, ${\eta }_{p }^{2}=0.31$, with a bias for visual (0.12) and virtually no bias for auditory (0.01) duration stimuli. The main effect of interference task (*p* = 0.091) and the interaction between duration modality and interference task (*p* = 0.092) were not significant.

### Secondary task performance (color and pitch discrimination)

**Fig. 3:**
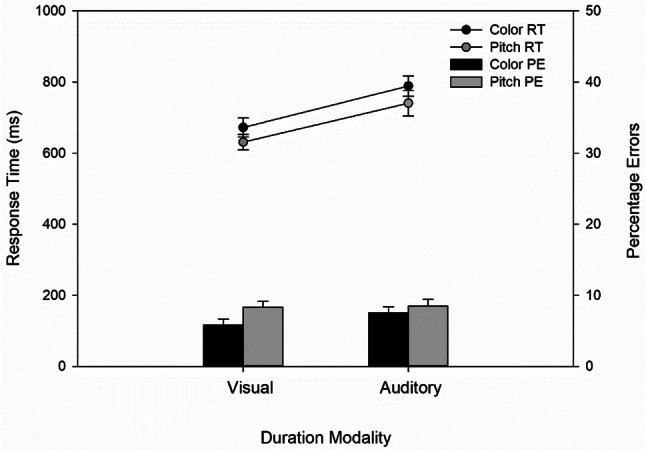
Mean response time (RT, lines) and percentage of errors (PE, bars) as a function of interference task and duration modality. Error bars represent  ± 1 within-subject error according to Morey (2008)

Figure 3 depicts mean RT and error rates as a function of interference task and duration modality. The analysis of mean RT revealed a main effect of duration modality, *F*(1, 47) = 10.69, *p* = 0.002, ${\eta }_{p }^{2}= 0.19$. Responses to the color or pitch change were faster for visual (651 ms) than for auditory (765 ms) duration stimuli. The main effect of interference task was not significant, *F*(1, 47) = 2.21, *p* = 0.144, ${\eta }_{p }^{2}= 0.01$. There was also no indication of any interaction between duration modality and interference task, *F*(1, 47) = 0.03, *p* = 0.854, ${\eta }_{p }^{2}< 0.01$. The analysis of error rates revealed no significant main or interaction effect, all *p*s ≥ 0.123. Secondary task performance thus shows a pattern slightly different to the one of primary task performance. Auditory duration discrimination impaired response speed in the secondary task, irrespective of the modality of the interference task^1^.

## Discussion

Previous studies regarding modality-specific interference effects in short-term retention of temporal information obtained discrepant results. Rattat and Picard (2012) observed modality-specific interference effects, that is, articulatory suppression selectively impaired retention of auditory durations and visuo-spatial tracking selectively impaired retention of visual durations. In contrast, Bratzke et al. (2016) observed a modality-independent interference effect of articulatory suppression on the retention of both, auditory and visual durations, whereas visuo-spatial tracking did not interfere with duration discrimination. The present results display a third pattern, which shares certain aspects of both previous result patterns. In line with Rattat and Picard’s results, auditory interference selectively impaired the retention of auditory durations. In contrast to their results, visual interference did not impair the retention of visual durations, a result that is consistent with Bratzke et al.’s previous results. Nevertheless, the present results do not replicate the crossmodal interference effect observed by Bratzke et al., that is, discrimination of visual durations was not affected by the auditory interference task. If one assumes that the visual interference task used in the present study was simply not effective as an interference task, these results seem to match best with the previous results by Rattat and Picard and to support their conclusion that short-term memory of temporal information is modality-specific.

This interim conclusion, however, needs to be reconsidered in light of possible trade-offs between primary task (duration discrimination) and secondary task (interference tasks) performance. Analysis of secondary task performance in the present study indeed revealed crossmodal interference, which did not show up in primary task performance. That is, the retention of auditory durations resulted in slower responses (compared with visual durations) in the interference task, both for auditory (pitch) and visual (color) signals. This crossmodal interference, however, cannot explain why the auditory interference task did not impair discrimination performance for visual durations. If one assumes that participants selectively traded off secondary task performance against primary task performance in this condition, one would have expected poorer pitch discrimination performance for the visual than for the auditory discrimination task. The results, however, showed the opposite pattern. Thus, the interim conclusion in favor of modality-specific short-term memory of temporal information still holds even in light of secondary task performance.

Given the present evidence for modality-specific interference, how can we explain the discrepant results by Bratzke et al. (2016) regarding the auditory interference condition? An obvious difference between the present and previous studies is the use of an auditory interference task other than articulatory suppression. Franssen, Vandierendonck and Van Hiel (2006) investigated timing performance with empty auditory intervals under a variety of concurrent auditory interference conditions, including articulatory suppression, irrelevant speech, irrelevant tones, and music. They observed that only the “active” articulatory suppression interfered with timing performance. It is conceivable that articulatory suppression and the present auditory interference task (pitch discrimination) affect different parts of the phonological loop, namely the subvocal rehearsal process and the phonological store, respectively (see also Franssen et al., 2006). Possibly, amodal and modal representations of temporal information coexist in short-term memory (see also Bratzke & Ulrich, 2019a; Stauffer et al., 2012) and the amodal representation can be disrupted by articulatory suppression whereas the modal representation can be disrupted by less “active” interference tasks like pitch discrimination in the present study. While these considerations are of course speculative and still cannot fully resolve the discrepancy of previous results, they highlight the importance of the present result that short-term memory of temporal information can be disrupted by an auditory interference task other than articulatory suppression.

In conclusion, previous results provided discrepant results regarding the modality specificity of interference effects between short-term retention of temporal information and modality-specific interference tasks. The present study aimed to resolve this discrepancy by analyzing possible trade-offs between primary and secondary task performance. The results are partly consistent with previous results by Rattat and Picard (2012) in favor of modality-specific short-term representations. Nevertheless, the present results suggest the possibility of coexisting amodal and modality-specific representations. Furthermore, the present study demonstrates that short-term memory of auditory durations can be disrupted by an interference task other than articulatory suppression.

## Footnotes

1 To account for potentially different speed-accuracy trade-offs within secondary task performance, we additionally analyzed the balanced integration score (BIS; Liesefeld & Janczyk, 2019), which integrates speed and accuracy. This analysis revealed essentially the same pattern as the RT analysis, with a significant main effect of duration modality, *F*(1, 47) = 10.78, *p* = 0.002, ${\eta }_{p }^{2}=$ 0.19, and no significant main effect of interference task or interaction effect, *p*s ≥ 0.422.
